# Obstetrics

**Published:** 1880-11

**Authors:** 


### OBSTETRICS.

A CASE OF SPONTANEOUS VERSION.—S. Lachapelle. (*L'Union Méd. de Can.*, p. 342, Aug. 1880.)

The author was called to attend a woman at term whose former labors had been natural and prompt. Examination showed the bag of waters broken, presentation of the vertex with occiput to the left sacro-iliac articulation, pelvis large, and considerable dilatation of the os. The bed was prepared and a second examination made: a strong pain, occiput to the sacrum. At the end of ten minutes a violent pain was felt by the woman, but without effect, upon the head, which did not advance. A few moments after, another pain so serious, that the head having almost completely passed the neck of the womb and resting upon the floor of the pelvis, I introduced the finger into the rectum, according to an old custom, to aid a prompt disengagement of the head. Suspension of pain; here I announced to the patient that the next pain would deliver her; another pain, no advancement, but the head retired rather. I awaited a new contraction, which came soon, and examination showed still greater receding of the head. Some minutes of repose, then a new contraction coming on, I examined, and my hand encountered the cord at the left descending *en masse;* then, more to the right, a foot, and to the extreme right, opposite the iliac crest, the head. To hesitate longer appeared to me to be dangerous, and seizing the foot, I effected the version without trouble, and the labor was soon terminated. Fearing a too strong contraction of the neck, I at once sought for the placenta, which I had great trouble in extracting on account of the resistance of the neck. There was grave hemorrhage, which, however, was not followed by any grave secondary complication.

Such is the case in all its nakedness, for though not complete, I have no doubt that it would have been if I had not interfered. What seems remarkable is, not the spontaneous version itself, for that must be admitted, but the spontaneous version after escape of the amniotic fluid, which many authors deny.

I avow, without shame, that I had not foreseen such a thing, for I was ignorant of the possibility of it, sharing the opinion of those who deny it. If it had not been for this ignorance on my part, I might have been able to study the course of the pains and prove if there was in this case irregular contraction of the walls of the uterus. It is in this way that spontaneous version must be explained. Cazeaux says: "I am led to believe that irregularity of contractions is not strange in such cases. In certain cases the organ seems not to contract except in a certain part of its extent, the other closing with less force or even remaining completely inert."

